# 611. No Source Control: Low Rates of Medication for Opioid Use Disorder in Individuals Hospitalized with Infectious Complications of Injection Opioid Use at Four Academic Medical Centers

**DOI:** 10.1093/ofid/ofab466.809

**Published:** 2021-12-04

**Authors:** Elana S Rosenthal, Jillian S Catalanotti, Christopher J Brokus, Joseph Carpenter, Ellen Eaton, Ellen Eaton, Irene Kuo, Alaina Steck, Hana Akselrod, Kaylee W Burgan, Greer A Burkholder, Keanan McGonigle, William Mai, Melissa Notis, Junfeng Sun, Sarah Kattakuzhy

**Affiliations:** 1 University of Maryland, Washington, DC; 2 The George Washington University of Medicine and Health Sciences, Washington, DC; 3 University of Maryland School of Medicine, Boston, MA; 4 Emory University School of Medicine, Atlanta, Georgia; 5 University of Alabama at Birmingham, Birmingham, Alabama; 6 George Washington University Milken Institute School of Public Health, Washington, DC; 7 University of Alabama Birmingham, Birmingham, Alabama; 8 CCMD/NIH, Bethesda, Maryland

## Abstract

**Background:**

Rates of hospitalization for bacterial infections due to opioid use disorder (OUD) are rising. Medication for OUD (MOUD) is an evidence-based intervention to treat OUD; however, MOUD initiation during hospitalization remain suboptimal. We aim to understand the continuum of MOUD and impact of MOUD initiation on outcomes of patients hospitalized with infectious complications of OUD.

**Methods:**

CHOICE is a retrospective review of adults hospitalized with an infectious complication of OUD and IDU at four academic medical centers (Figure 1). Patients were hospitalized between 1/1/2018 and 12/31/2018, had ICD9/10 diagnosis codes consistent with OUD and acute bacterial/fungal infection, and chart review verification of active infection associated with OUD. Data were abstracted regarding demographics, inpatient interventions, transitions of care, and 1 year outcomes. Linear regression model with generalized estimating equation was used to evaluate associations of MOUD initiation with outcomes.

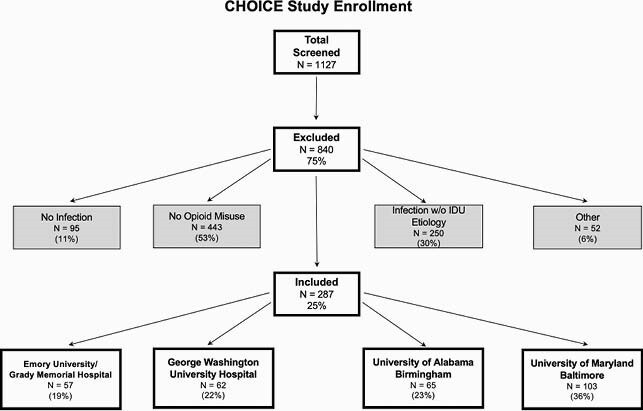

**Results:**

287 patients were predominately male (59%), white (63%), and median age 40 (32;52), with 72 (25%) uninsured, 103 (36%) unstably housed, and 84 (29%) were on MOUD prior to admission. 129 (45%) received MOUD during admission, 113 (39%) had MOUD prescribed on discharge, and 24 (8.4%) were linked to MOUD after admission [fig 2]. During sentinel admission, 62 (22%) were discharged prematurely/eloped, of whom 43 (69%) left without an antibiotic plan. Of the 202 (71%) not on MOUD at baseline, 55 (27%) initiated MOUD during admission. MOUD initiation was associated with higher odds of planned discharge (OR 6.7; p=0.0002) and being discharged on MOUD (OR 174; p< 0.0001) [fig 3]. Being uninsured was associated with lower odds of planned discharge (OR 0.55; < 0.0001) and discharge on MOUD (OR 0.59; p=0.02).

CHOICE Baseline Demographics (N=287)

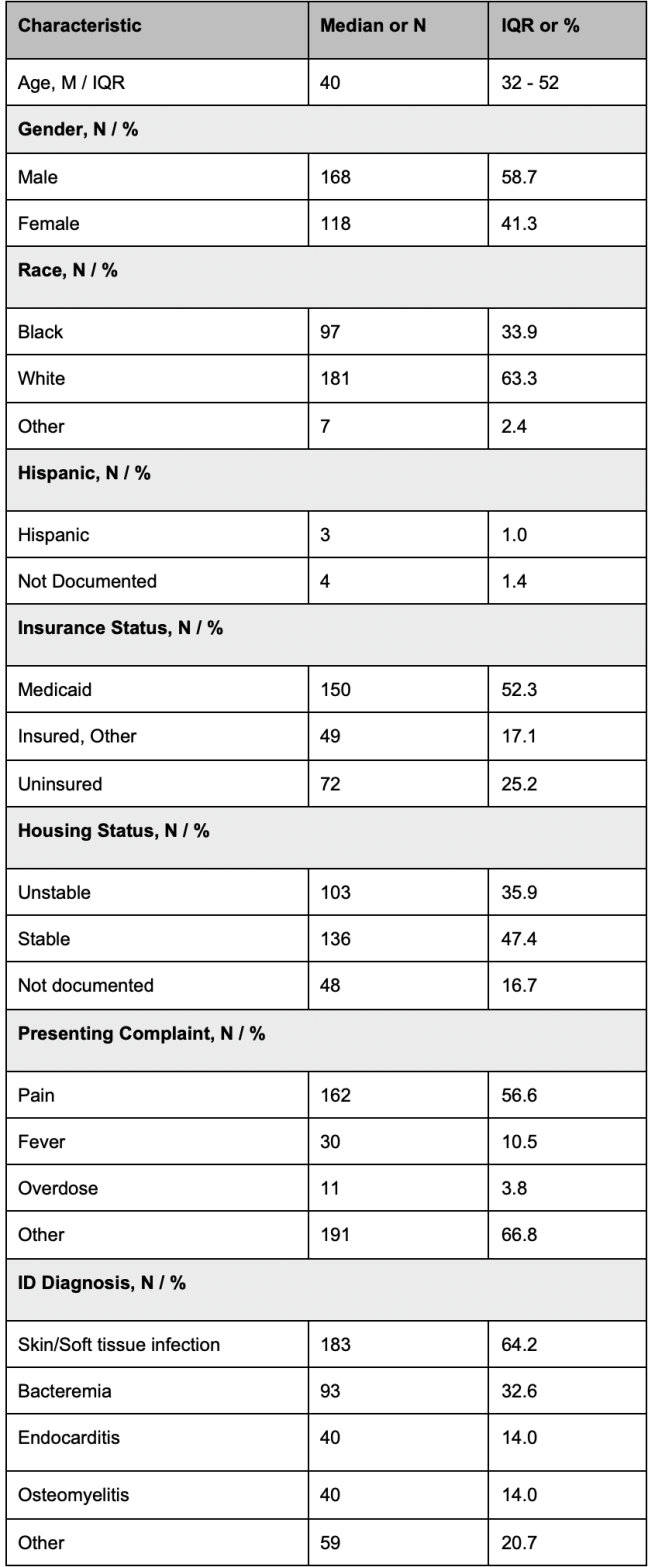

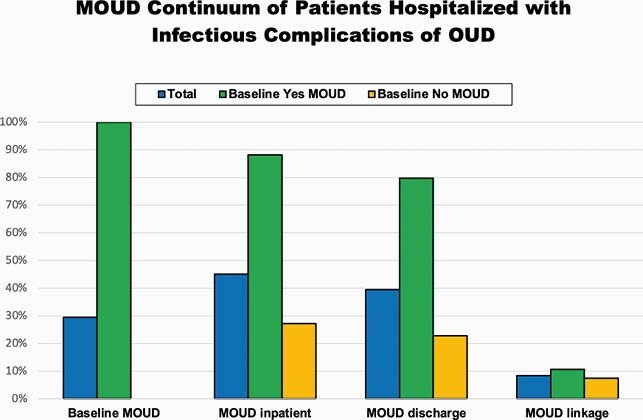

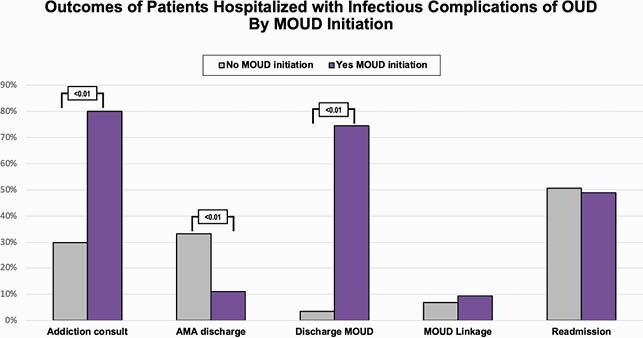

**Conclusion:**

Across four healthcare systems, we found that patients hospitalized with infectious complications of OUD had low rates of MOUD initiation and high rates of premature discharge with incomplete ID treatment. Interventions to increase MOUD initiation and expand access to insurance may serve to mitigate the morbidity and mortality associated with OUD-related infections.

**Disclosures:**

**Elana S. Rosenthal, MD**, **Gilead Sciences** (Research Grant or Support)**Merck** (Research Grant or Support) **Ellen Eaton, MD** , **Gilead** (Grant/Research Support) **Ellen Eaton, MD** , Gilead (Individual(s) Involved: Self): Research Grant or Support **Greer A. Burkholder, MD, MSPH**, **Eli Lilly** (Grant/Research Support) **Sarah Kattakuzhy, MD**, **Gilead Sciences** (Scientific Research Study Investigator, Research Grant or Support)

